# Objective assessment of dysarthric disorders in patients with multiple sclerosis depending on sex, age, and type of text read

**DOI:** 10.3389/fneur.2023.1225754

**Published:** 2023-08-09

**Authors:** Wojciech A. Warmbier, Małgorzata Popiel, Agnieszka Guzik, Mariusz Drużbicki, Halina Bartosik-Psujek

**Affiliations:** ^1^BD Center Ltd., Rzeszow, Poland; ^2^Clinical Hospital No 2 in Rzeszow, Rzeszow, Poland; ^3^Subcarpathian Center for Neurorehabilitation, Rzeszow, Poland; ^4^Department of Physiotherapy, Institute of Health Sciences, College of Medical Sciences, University of Rzeszów, Rzeszow, Poland; ^5^Department of Neurology, Institute of Medical Sciences, College of Medical Sciences, University of Rzeszów, Rzeszow, Poland

**Keywords:** multiple sclerosis, speech, dysarthria, objective tool, sex, age

## Abstract

**Purpose:**

To assess dysarthric disorders in multiple sclerosis (MS) patients in comparison with healthy individuals and MS patients without dysarthria depending on the patient’s sex, age, and the type of text read using an objective tool.

**Methods:**

The study was carried out in a group of 72 persons, including 24 with MS presenting dysarthria (study group) and 24 healthy individuals (healthy control group), and 24 with MS without dysarthria (MS control group). Performance (reading) time was evaluated by means of an objective tool created for the purpose of the analysis.

**Results:**

The study showed significant statistical differences in the analyzed performance time of: poetry reading, prose reading, and completing a diction exercise, among persons with MS from the study group presenting dysarthria and both control groups (*p* < 0.05). It took more time to read the poem, and prose and to perform a diction exercise in the study group with dysarthria than in both control groups (with no significant differences between the two) Similarly, the comparison between the groups in terms of sex and age showed disturbances in the above-mentioned parameter in the study group. What was not demonstrated were significant differences in the evaluated speech parameters depending on both sex and age separately in the group of MS patients with dysarthria, and both control groups (*p* < 0.05).

**Conclusion:**

The objective tool created for the purpose of speech analysis is useful in detecting discrepancies in performance (reading) time among MS patients with dysarthria, and healthy individuals, as well as patients with MS without dysarthria and can be used in clinical practice for diagnostic purposes, however, further research is essential to complete its validation.

## 1. Introduction

Multiple sclerosis (MS) is an immunological, inflammatory disease demyelinating the central nervous system, which constitutes the most common nontraumatic cause of disabilities in young adults (1). Currently, a total of 2.8 million people is estimated to live with MS worldwide, 35.9 per 100,000 population (2). MS prevalence has increased in every world region since 2013 (2). The newest data indicate that MS incidence and prevalence in Poland are higher than previously reported and in 2019 amounted to 6.6 and 131.2/100,000 inhabitants, respectively (3).

The onset of MS usually occurs between the ages of 20 and 40 and is two to three times more common in women than men. Most commonly the disease is of relapsing–remitting type (85–90%), progressing over time. A small percentage of patients (10%) are diagnosed with primary progressive MS, which is characterized by progression from the onset of the disease (4).

In the relapsing–remitting form, each relapse may be associated with a different type of neurological deficit. Typical clinical symptoms include retrobulbar inflammation of the optic nerve, eye movement disorders, cerebellar ataxia, spastic paresis, and sensory disturbances (5). Pyramidal pathways damage is the cause of paresis of the limbs, increased muscle tone, exaggerated deep reflexes, and the presence of pathological symptoms. In the initial phase of the disease, these symptoms occur in 32–41% of patients, and in the majority of MS patients (90%) in the chronic phase. Cerebellar ataxia stems from dysfunction in the cerebellum, resulting in uncoordinated movements whereas sensory ataxia arises due to the impairment of sensory input in regulating movement. The symptoms of the cerebellar syndrome include: dysarthria, dysmetria, dysadiadiochokinesis, intention tremor, dysrhythmia, disturbances in motor coordination and balance. Unlike cerebellar ataxia sensory ataxia (damage to the dorsal columns) is not accompanied by dysarthria, and nystagmus, or postural abnormalities, but impairment in deep sensation, attenuation or loss of deep reflexes, and finger-nose test worsening with eyes closed (proprioception deficit) (6). Among the study group, symptomatically, all subjects were characterized by features of atactic disorders and pyramidal signs. In 8.33% of the study group, additional disorders associated with cranial nerves were observed.

The combination of neurological symptoms can be extremely varied and variable. Speech and voice disorders are among the least accurately described clinical symptoms of MS, although their estimated prevalence reaches 40–50% (7–9). Demyelinating damage to the central nervous system may cause spasticity, weakness of the tongue muscles, and impaired motor coordination of the tongue, jaw, soft palate, vocal cords, and diaphragm (10). Communication impairment may result from difficulties in voice control and articulation of words due to the dysfunction of the speech-responsible muscles and insufficient subglottic pressure (11). The most commonly reported speech disorders include speech, speech speed reduction, voice quality deterioration, hoarseness, volume and tone control disorders, imprecise articulation, impaired speech fluency, and swallowing problems (10, 12, 13). Speech disorders in patients with MS are associated with negative physical and psychosocial consequences, including communication problems, frustration, low self-esteem, and limited participation in daily activities (14). Studies show that dysarthria, i.e., a motor disorder of speech function arising as a result of sudden as well as chronic diseases causing problems with effective verbal communication, is the most common speech disorder in MS, affecting up to 45% of patients (15). There are many types of dysarthria: cerebellar, spastic, bulbar, and dystonic. In the case of MS when the disorder is present it can mirror either a single type or a mixture of a few types, with spastic-ataxic being the most frequent. Spastic dysarthria is a combination of weakness and spasticity. It can manifest itself with slow and reduced range and force of speech. Ataxic dysarthria is associated with damage to the cerebellar control circuit. Associated with disturbed coordination, it may occur in all speech levels: respiration, phonation, resonation, and articulation, however, it is most noticeable in terms of articulation and prosody (16). For our research we chose a group presenting mixed, spastic-ataxic dysarthria. The study of these disorders can (especially in the context of tracking the dynamics of changes) become an effective and accurate diagnostic tool, especially in chronic diseases whose complications include neuromuscular disorders (15).

So far, in Poland, the deficits have been commonly assessed on the basis of an interview collected from patients (17). The Speech Pathology Specific Questionnaire for people with MS has also been developed and validated (7). Based on the review of the world literature, it can be concluded that in many countries the following scales are most often used in phoniatric practice: Vocal Tract Discomfort, GRBAS listening scale, Voice Handicap Index (18–21). All of the above-mentioned scales are subjective tools, and therefore they are burdened with a certain margin of error, thirdly, they do not allow for the unequivocal distinction between physiological and pathological values, therefore they are of limited use in routine clinical examinations of the vocal organ and do not accentuate on the mechanisms inducing the phenomenon of dysarthria, which is crucial in conducting therapy (22).

The observations became the motivator for undertaking the discussed research. Another reason was the fact that researchers currently report there are no MS-specific diagnostic instruments for dysarthria (15, 23). Providing there is an objective tool for measuring speech deterioration and its sensitivity to disease activity is confirmed in the studies, dysarthria may, in the future, be used as a biomarker for the progression of the disease (15). Therefore, we decided to create an objective tool to assess dysarthric disorders in patients with MS.

The purpose of the study was to assess dysarthric disorders in MS patients in comparison with healthy individuals and MS patients without dysarthria depending on the patient’s sex, age, and the type of text read using an objective tool.

## 2. Materials and methods

### 2.1. Participants and setting

The study was carried out in the Clinical Neurology Ward with Stroke Unit at the Clinical Hospital No 2 in Rzeszow, Poland, as a part of broader research analyzing speech parameters of native speakers of Polish suffering from MS. It was conducted in a group of 72 persons, including 24 with multiple sclerosis (MS) presenting mixed spastic-ataxic dysarthria (study group), 24 healthy individuals (healthy control group) and 24 persons with MS without dysarthria (MS control group). The patients were treated for their MS in the Clinical Neurology Ward with Stroke Unit at the Clinical Hospital No 2 in Rzeszow, Poland, as part of the government-funded pharmacological treatment. The study group comprised 12 women and 12 men, with a mean age of 39.2 ± 12.30. Both control groups were age- and sex-matched to the study group. The characteristics of the three groups are shown in Table 1.

**Table 1:** Baseline characteristics of study and two control groups.

	Study group (*N* = 24)	MS control group (*N* = 24)	Healthy control group (*N* = 24)
Age [years], mean (SD)	39.2 (12.3)		41.25 (12)
Sex [female/male], N	12/12	12/12	12/12
Age up to 40 years, N	12	12	12
Age below 40 years, N	12	12	12
Time from MS [years], mean (SD)	12.08	9.95	-
EDSS, mean (SD)	4.29 (1.62)	3.45 (1.77)	-
MS type, %	RR 54.16 SP 37.5 PP 8.33	RR 58.33 SP 29.17 PP 12.5	-
Level of education, %	Secondary 83.33 Higher 16.67	Secondary 79,17 Higher 20.83	Secondary 83.33 Higher 16.67

N, number of subjects; SD, standard deviation; MS, multiple sclerosis; EDSS, Expanded Disability Status Scale; RR, relapsing–remitting; SP, secondary progressive; PP, primary progressive.

The study group included people diagnosed with MS, presenting with mixed, spastic-ataxic dysarthria, in remission, who gave their informed consent to participate in the study. Patients with cognitive deficits impairing the ability to understand and follow instructions (Mini-Mental State Examination <24), with visual impairment, and those: with speech disorders other than spastic-ataxic dysarthria, or other than dysarthria; in the period of relapse; with any comorbidities that may affect the quality of speech; or who did not consent to participate in the study were excluded. The MS control group included people diagnosed with MS without dysarthria or any speech disorders, matched in terms of age and sex. The healthy control group consisted of healthy people, without any speech disorders, matched in terms of age and sex.

The study protocol was assessed and accepted by the Bioethical Committee at the University of Rzeszów (approval no. 3/01/2020). All the procedures were executed in full compliance with the principles set forth in the Declaration of Helsinki. All the study participants gave their informed consent in writing.

### 2.2. Procedures

The following work implements an analysis of performance time based on recording samples of the study participants reading three suggested texts: (1) a poem, (2) a text in prose, and (3) a one-line diction exercise. Every participant had time to read the text before the recording began. Only when the examiner made sure that everything in the text was understandable and that the subject was ready to read it out loud, did the recording start.

(1) A poetic text: 16 lines 10 syllables each – a poem in verse 2 lines each;
(2) A text in prose: including simple and complex sentences containing all sounds of the Polish language, as well as phonetic phenomena indicating potential dysarthric disorders, for instance, consonantal clusters provoking phonetic mistakes.

4 sentences, 3 complex and 1 simple: 1st sentence – 24 words, 2nd sentence – 4 words, 3rd sentence – 21 words, 4th sentence – 23 words;

(3) The diction exercise: required accurate (without phonetic mistakes) realization of 10 syllables in the fastest possible manner.

PA TA KA PA TA KA PA TA KA PA.

All samples qualified for the study were then assessed by a specialist in terms of the severity of the disorder considering the performance time (PT) of each text in seconds.

### 2.3. Outcome measures

For the purposes of this study, an objective speech analysis tool (an IT tool, concerning information technology, software with elements of machine learning) was created. The neural network model was implemented based on the TensorFlow library.

#### 2.3.1. Layers of the model

The Model consists of 19 layers of a total number of 572,868 parameters (including 572,100 that undergo the learning process). Eleven layers, created in line with the Convolutional Neural Network (CNN), comprise three blocks of the following spread:

Plexus → Plexus → Data Normalization → Subarea Maximum. Due to the fact that the sound samples vary in length the network that was used would be able to remember the previous state while ‘listening’ to the recordings. In a classic neural network, the so-called feed-forward neural network, the input data passes through the hidden layers, therefore the output data is determined. In a trained network, specific inputs always generate exactly the same outputs. In the case of a recursive network (RNN), an additional loopback was used to remember the state between successive calls. Input x may return different y values each time, depending on the currently stored state of the network h (hidden state). Classic RNN layers involve a gradient, which exponentially disappears in time while reverse learning – causes the older data to be forgotten quickly, with the newest data having the greatest impact on final results. The advantage of classic RNNs is that they learn faster and require fewer resources. Unfortunately, in this case, they did not give satisfactory effectiveness. For this reason, the LSTM (Long Short-Term Memory) architecture was used. If we treat the LSTM cell as a black box, it is similar to a classic recursive cell, except that its state is divided into two vectors: h (hidden, storing short-term data) and c (cell, storing long-term data).

#### 2.3.2. Activation function

**Figure 1:**
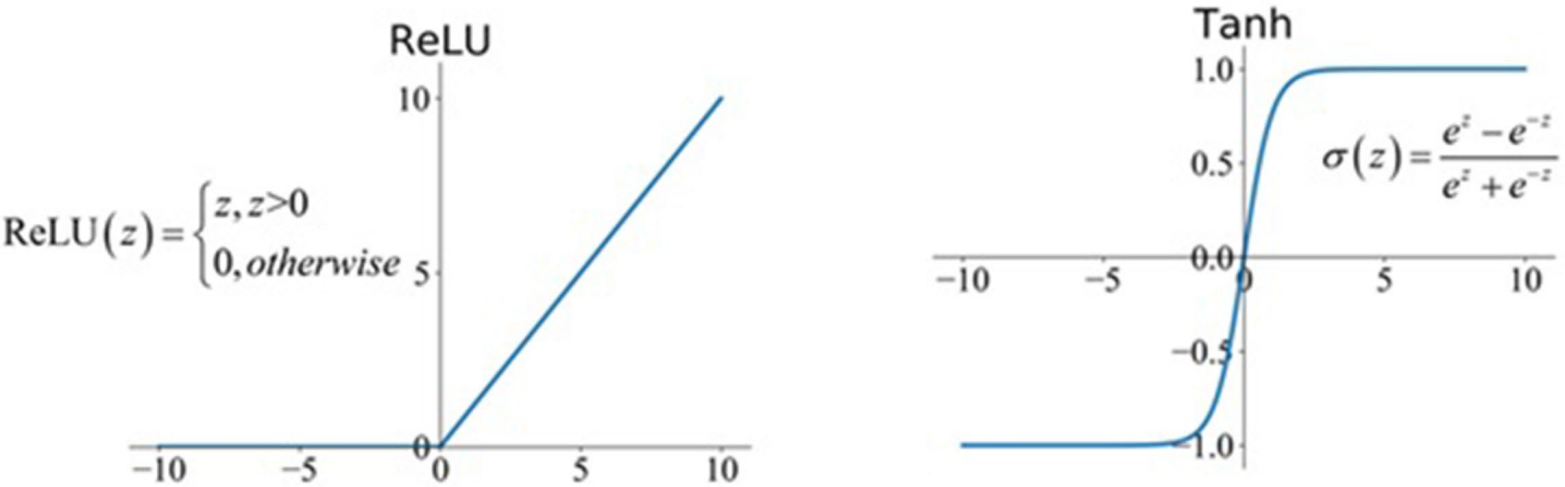
The activation function in the network model.

In the model, the networks used include the following activation functions (Figure 1):

- ReLU, for positive values behaves like a linear function and returns as 0 for negative values. As it is a nonlinear function, it works well in hidden layers and is not used in output layers. It was applied in all hidden layers of the convolutional neural network.
- Tanh, is similar to the sigmoid function, however, its core constitutes 0 and covers a bigger area. It is quite flat for large values so it still can cause slow network learning. It was used in hidden layers in the recurrent parts (RNN).
- Softmax returns probabilities of belonging to disjoint classes. The values are normalized (the sum of probabilities equals 1.0). It is a milder version of the argmax function, which outputs the highest value index. It was used in the output layer to determine the probability of receiving a particular evaluation.

#### 2.3.3. Activation thresholds

In each one of the neurons, all input signals are multiplied by individual weights of input signals and compared to the activation threshold. Reaching the activation threshold results in evoking the following neurons in the consecutive network layers.

#### 2.3.4. Network learning process

Neural network underwent a learning process that is based on a proper selection of weight factors based on the examination of breathing and phonation disorders. Therefore, it was essential to find the best method and algorithm to effectively conduct the process. Training data were set by Short-Time Fourier Transform (STFT) in the form of binary samples. For learning, the network uses a backpropagation algorithm, which proved perfectly in the controlled teaching process of a multi-layered one-directional neural network. The name of the algorithm originates from the order of counting error d signals, which runs in the opposite direction to the way the signals travel within the network, from the input layer through the hidden layers to the output layers. The algorithm allowed to count gradients, the direction in which the error is minimalized. The value charges the first-row optimizer, which based on gradient analysis corrects weights in the model. Adam’s optimization method was applied as the first-row optimizer. The algorithm was first described by Diederik et al. (24).

### 2.4. Data analyses

The analysis was performed in the R program, version 4.2.2. Quantitative variables (i.e., expressed in numbers) were analyzed by calculating the mean, standard deviation, median, and quartiles. ANOVA (followed by Fisher’s LSD *post-hoc* test) was used to compare quantitative variables between the three groups. The relationship between two quantitative variables was assessed with Pearson’s coefficient of correlation. The analysis adopted a significance level of 0.05. Thus, all *p*-values below 0.05 were interpreted as significant associations.

## 3. Results

### 3.1. Comparison within the groups: sex and age

On analyzing the performance time (PT) of the three suggested texts: (1) poem [s], (2) a text in prose [s], and (3) a diction exercise [s] between women and men separately in the study group and in both control groups no statistically significant differences (*p* > 0.05). The PT parameter was then compared between the age groups: 20–40 and 40–62 among women and men in the study group and both control groups with no statistically significant differences found (*p* > 0.05). Similarly, in the corresponding age groups, i.e., 20–40 and 40–62, between women and men separately in the study group and in both control groups no statistically significant differences were found (*p* > 0.05).

### 3.2. Comparison between the groups

#### 3.2.1. Study group to control group

Statistically significant differences were found (*p* < 0.05) in the performance time (PT) of people from the study group and both control groups. Reading a poem, reading prose, and completing the diction exercise took more time in the dysarthria study group than in both control groups, which did not differ significantly (Table 2).

**Table 2:** Comparison between the groups.

Text	Group	*N*	Mean	SD	Median	Min	Max	Q1	Q3	*p*
Poem [s]	Study group	24	57.24	23.66	53.44	35.55	153.97	44.37	58.25	*p* < 0.001*
	Healthy Control group	24	36.21	4.97	36.72	28.33	44.44	31.61	38.76	
	MS Control group	24	38.35	6.94	36.94	29.86	65.88	34.86	40.57	S > C,CMS
Prose [s]	Study group	24	79.90	32.14	71.34	53.63	209.26	61.42	90.82	*p* < 0.001*
	Healthy Control group	24	46.40	8.77	46.23	32.89	66.02	40.24	50.47	
	MS Control group	24	47.05	7.60	46.19	35.03	62.01	41.48	50.40	S > C,CMS
Diction exercise [s]	Study group	24	3.17	1.12	2.86	1.97	6.11	2.31	3.78	*p* < 0.001*
	Healthy Control group	24	1.67	0.45	1.58	1.08	2.92	1.38	1.85	
	MS Control group	24	1.69	0.36	1.64	1.07	2.76	1.48	1.80	S > C,CMS

*p*, ANOVA + Fisher’s LSD *post-hoc* test; SD, standard deviation; Q1, lower quartile; Q3, upper quartile. *statistically significant difference (*p* < 0.05). S, study group; C, healthy control group; CMS, MS control group.

#### 3.2.2. Study group to control group: sex and age

In terms of analyzing differences in PT between women from the study group and both control groups, and men from the study group and both control groups, statistically significant differences were found in all instances (*p* < 0.05). Reading a poem, reading prose, and completing the diction exercise took more time in the dysarthria study group than in both control groups, which did not differ significantly (Table 3).

**Table 3:** Comparison between the groups in terms of sex.

Text	Sex-Group	*N*	Mean	SD	Median	Min	Max	Q1	Q3	*p*
Poem [s]	Women in the study group	12	51.94	14.80	49.99	35.55	89.32	42.11	58.25	*p* = 0.001*
	Women in the healthy control group	12	36.51	5.64	36.53	28.96	44.44	30.83	41.45	
	Women in the MS control group	12	37.85	4.70	37.56	29.86	45.54	34.79	41.70	S > C,CMS
Prose [s]	Women in the study group	12	72.98	17.35	67.47	53.63	108.36	58.84	84.20	*p* < 0.001*
	Women in the healthy control group	12	46.60	8.27	46.23	34.34	62.48	40.87	50.35	
	Women in the MS control group	12	48.27	7.76	46.46	37.78	61.87	44.33	52.98	S > C,CMS
Diction exercise [s]	Women in the study group	12	3.18	0.83	3.09	1.97	4.55	2.54	3.87	*p* < 0.001*
	Women in the healthy control group	12	1.77	0.55	1.58	1.20	2.92	1.42	1.98	
	Women in the MS control group	12	1.82	0.42	1.76	1.37	2.76	1.55	1.82	S > C,CMS
Poem [s]	Men in the study group	12	62.55	29.83	53.82	42.25	153.97	50.67	59.71	*p* = 0.002*
	Men in the healthy control group	12	35.91	4.42	37.24	28.33	42.25	33.23	37.96	
	Men in the MS control group	12	38.85	8.84	36.76	32.59	65.88	34.98	37.39	S > C,CMS
Prose [s]	Men in the study group	12	86.81	41.88	74.92	54.39	209.26	65.08	93.52	*p* < 0.001*
	Men in the healthy control group	12	46.21	9.62	45.02	32.89	66.02	40.24	50.82	
	Men in the MS control group	12	45.82	7.57	44.47	35.03	62.01	41.29	48.19	S > C,CMS
Diction exercise [s]	Men in the study group	12	3.15	1.39	2.54	2.05	6.11	2.23	3.35	*p* < 0.001*
	Men in the healthy control group	12	1.58	0.32	1.56	1.08	2.03	1.37	1.85	
	Men in the MS control group	12	1.56	0.24	1.59	1.07	1.95	1.41	1.68	S > C,CMS

*p*, ANOVA + Fisher’s LSD *post-hoc* test; SD, standard deviation; Q1, lower quartile; Q3, upper quartile. *statistically significant difference (*p* < 0.05). S, study group; C, healthy control group; CMS, MS control group.

Similarly, in the analysis of performance time between women in the 1st age group of the study and women in the 1st age group of both control groups, statistically significant differences were found in all analyzed speech parameters (*p* < 0.05). Poem reading, prose reading, and diction exercise took longer in the study group than in both control groups, which did not differ significantly. Statistically significant differences (*p* < 0.05) were found between women in the 2nd age group of the study group and women in the 2nd age group of both control groups (*p* < 0.05) in terms of reading prose and performing diction exercises, which took more time in the study group (Table 4).

**Table 4:** Age comparison between the groups: women.

Text	Sex-Group-Age group	*N*	Mean	SD	Median	Min	Max	Q1	Q3	*p*
Poem [s]	Women-Study group-1st age group	6	48.57	10.29	49.49	35.55	59.82	40.38	57.15	*p* = 0.007*
	Women- Healthy Control group-1st age group	6	33.77	4.77	32.57	28.96	40.82	30.16	36.90	
	Women- MS Control group-1st age group	6	37.54	4.82	38.49	29.86	42.35	35.01	41.28	S > C,CMS
Prose [s]	Women-Study group-1st age group	6	67.50	15.51	60.62	53.63	91.82	57.20	76.84	*p* = 0.003*
	Women- Healthy Control group-1st age group	6	42.54	5.70	43.44	34.34	49.51	38.81	46.27	
	Women- MS Control group-1st age group	6	48.38	8.51	48.30	37.78	61.87	42.86	51.81	S > C,CMS
Diction exercise [s]	Women-Study group-1st age group	6	3.26	0.82	3.40	2.19	4.06	2.61	3.98	*p* < 0.001*
	Women- Healthy Control group-1st age group	6	1.50	0.22	1.48	1.20	1.84	1.38	1.62	
	Women- MS Control group-1st age group	6	1.84	0.47	1.67	1.49	2.76	1.58	1.83	S > C,CMS
Poem [s]	Women-Study group-2nd age group	6	55.32	18.67	51.53	37.43	89.32	43.70	58.67	*p* = 0.037*
	Women- Healthy Control group-2nd age group	6	39.25	5.40	39.97	31.00	44.44	36.49	43.56	
	Women- MS Control group-2nd age group	6	38.16	5.01	37.56	31.44	45.54	35.44	40.97	S > C,CMS
Prose [s]	Women-Study group-2nd age group	6	78.46	18.68	72.28	58.75	108.36	66.80	88.66	*p* = 0.001*
	Women -Healthy Control group-2nd age group	6	50.67	8.84	49.83	38.20	62.48	46.06	56.64	
	Women- MS Control group-2nd age group	6	48.16	7.73	45.79	37.93	59.80	45.34	52.31	S > C,CMS
Diction exercise [s]	Women-Study group-2nd age group	6	3.11	0.91	2.96	1.97	4.55	2.60	3.52	*p* = 0.012*
	Women-Healthy Control group-2nd age group	6	2.03	0.67	2.04	1.24	2.92	1.51	2.48	
	Women- MS Control group-2nd age group	6	1.81	0.42	1.79	1.37	2.57	1.57	1.81	S > C,CMS

*p*, ANOVA + Fisher’s LSD *post-hoc* test; SD, standard deviation; Q1, lower quartile; Q3, upper quartile. *statistically significant difference (*p* < 0.05). S, study group; C, healthy control group; CMS, MS control group.

In the analysis of differences in performance time between men in the 1st age group of the study group and men in the 1st age group of both control groups, statistically significant differences were found in all analyzed speech parameters (*p* < 0.05). Poem reading, prose reading, and diction exercise took longer in the study group than in both control groups, which did not differ significantly. Statistically significant differences were found between men in the 2nd age group in the study group and men in the corresponding 2nd age group of both control groups (*p* < 0.05). Poem reading, prose reading, and a diction exercise took longer in the study group (Table 5).

**Table 5:** Age comparison between the groups: men.

Text	Sex-Group-Age group	*N*	Mean	SD	Median	Min	Max	Q1	Q3	*p*
Poem [s]	Men-Study group-1st age group	6	70.95	41.25	55.01	44.30	153.97	52.29	63.30	*p* = 0.035*
	Men- Healthy Control group-1st age group	6	36.93	3.44	37.25	31.82	42.18	35.46	37.96	
	Men- MS Control group-1st age group	6	35.36	1.94	35.86	32.59	37.38	33.99	36.81	S > C.CMS
Prose [s]	Men-Study group-1st age group	6	95.15	58.33	70.97	54.39	209.26	63.72	95.76	*p* = 0.028*
	Men- Healthy Control group-1st age group	6	44.65	7.23	44.79	34.10	54.24	40.76	49.12	
	Men- MS Control group-1st age group	6	42.64	4.73	42.16	35.03	48.76	41.46	45.41	S > C,CMS
Diction exercise [s]	Men-Study group-1st age group	6	3.65	1.81	2.89	2.05	6.11	2.31	5.10	*p* = 0.004*
	Men- Healthy Control group-1st age group	6	1.54	0.32	1.56	1.08	1.87	1.34	1.80	
	Men- MS Control group-1st age group	6	1.40	0.20	1.40	1.07	1.65	1.34	1.53	S > C,CMS
Poem [s]	Men-Study group-2nd age group	6	54.14	9.30	53.38	42.25	69.49	49.17	57.26	*p* = 0.009*
	Men- Healthy Control group-2nd age group	6	34.90	5.36	35.67	28.33	42.25	30.60	37.85	
	Men- MS Control group-2nd age group	6	42.33	11.79	37.36	34.72	65.88	36.75	40.93	S > C,CMS
Prose [s]	Men-Study group-2nd age group	6	78.48	17.01	78.36	54.99	102.64	68.51	88.05	*p* = 0.001*
	Men- Healthy Control group-2nd age group	6	47.76	12.06	45.31	32.89	66.02	40.67	54.77	
	Men- MS Control group-2nd age group	6	49.01	8.91	47.66	38.91	62.01	42.64	54.54	S > C,CMS
Diction exercise [s]	Men-Study group-2nd age group	6	2.66	0.64	2.43	2.05	3.77	2.26	2.89	*p* = 0.001*
	Men- Healthy Control group-2nd age group	6	1.62	0.35	1.54	1.20	2.03	1.39	1.92	
	Men- MS Control group-2nd age group	6	1.71	0.17	1.69	1.46	1.95	1.63	1.81	S > C,CMS

*p*, ANOVA + Fisher’s LSD *post-hoc* test; SD, standard deviation; Q1, lower quartile; Q3, upper quartile. *statistically significant difference (*p* < 0.05). S, study group; C, healthy control group; CMS, MS control group.

### 3.3. Reference to the Expanded Disability Status Scale

Considering the relationship between the performance time of reading a poem, reading prose, and completing the diction exercise and the Expanded Disability Status Scale (EDSS) level in the study group no significant dependencies were found (*p* > 0.05) for each of the analyzed speech parameters (Table 6).

**Table 6:** Reference to the Expanded Disability Status Scale.

Text Study group	EDSS
	Pearson’s correlation coefficient
Poem [s]	*r* = 0.126, *p* = 0.556
Prose [s]	*r* = 0.284, *p* = 0.179
Diction exercise [s]	*r* = 0.128, *p* = 0.552

EDSS, Expanded Disability Status Scale.

## 4. Discussion

A review of the literature focusing on the issues shows that research evaluating the character of speech disorders in MS is limited (15, 23). The available dysarthria scales are based on more or less subjective data, which are more difficult to compare (15, 18–23). As noted in the review article by Noffs et al. objective speech assessment is more accurate, replicable, and feasible when contrasted with perceptual analysis (25) which occurs in the majority of the studies concerning the notion of dysarthria in the course of neurological diseases. Therefore our study focused on an objective assessment of speech features in dysarthric disorders in MS patients in comparison with healthy individuals depending on sex, age, and type of text read using an objective tool. The reports of Hartelius et al., who attempted a subjective assessment of speech difficulties in people with various types and degrees of dysarthria involved a self-report questionnaire, Living with Neurologically Based Speech Difficulties (Living with Dysarthria) (26). As in our studies, the authors showed that the degree of communication difficulties was not dependent on age and sex, and the dominant speech difficulties were associated with reduced speech speed and the need for repetition as a consequence of misunderstandings in communication with other people (26). Therefore, it can be assumed that sex and age do not differentiate dysarthric speech disorders, regardless of their cause.

We were unable to find publications that would allow a discussion between the results of the study and their verification concerning the analysis we performed that focused on the differences in speech speed rate during reading particular texts in Polish: a poem, a text in prose, and a diction exercise between women and men, separately, in the study group and in both control groups: healthy controls and MS without dysarthria controls.

As far as the analysis of performance time (PT) depending on the type of text read between the study group and both control groups is concerned statistically significant differences (*p* < 0.05) were observed. It is worth mentioning that all of the parameters were substantially higher in the study than in both control groups with no significant differences between them. Again, it was impossible to find published sources that would allow us to compare the obtained results with other authors.

In studies by Rusz et al. and Rodgers et al. the analysis of speech rate and acoustic, conducted by speech therapists’ rate was based on passage reading (11 sentences, 80 words) and on repetition of syllables/pa ta ka/and (puh puh kuh). The participants were asked to produce as many syllables as possible (minimum 7) per breath (8, 27). Then, the average number of syllables produced per second was studied. In our opinion making patients inhale a maximum of air changes the nature of speech production and increases the risk of biased results. We measured the time of producing 10 syllables without provoking the patients to make a disproportionate effort needed to complete the task, which in our view, is more reliable and easier to interpret by a therapist in the context of the diagnosis of dysarthria as well as potential evaluation of the dynamics of its progression. Moreover, when contrasted with speech rate, determining performance time, that is, the total time needed to read each text, is in this case a less demanding task, including an easier analysis process. Speech rate is a unit expressed by a count of words per minute or syllables per second, whereas performance time in seconds. we tested the dependencies between the prolonged performance time of the diction exercise between the study group and the control group. We did not observe any significant differences. The proportions were identical. When considering the diction exercise, in the study group the average SR (speech rate) amounted to 3.15 syllables/s and the average PT (performance time) was 3.17 s. In the control group of healthy individuals, the average SR was 6.18 syllables/s, and the average PT – 1.62 s. The execution time of the exercise by the dysarthric patients in the study group was longer by 96.2% when the speech rate was counted, and 95.7% longer when the performance time was measured. We believe that speech speed expresses as PT is a fundamental parameter differentiating correct speech from dysarthric.

The results of the aforementioned studies cannot be compared to ours due to the phonetic variety of the languages (English/Czech) and the unit used – we concentrate on the performance time of producing 10 syllables of the diction exercise and total reading time of the suggested text. The cited studies (8, 27) analyzed speech rate and articulation rate measured as words/min and syllable/s. There were no studies thar conducted assessment of the differentiation of reading of a poetic text.

A few studies comparing speech differences, were concerned with other groups of patients, i.e., adults with cerebral palsy being compared to healthy people (28). In their research, Liu and Chen tested both the question of whether consonant landmarks could be used as predictors for dysarthric speech in adult patients with cerebral palsy, as well as if there was a link between the aforementioned landmarks and the exacerbation of the speaking disorder. The researchers contrasted differences in the speech of seven adults with cerebral palsy suffering from dysarthria with the speech of seven healthy persons from the control group matched in terms of sex and age to the subjects from the study group (28). Similarly to our research, significant differences were observed between the subjects from the study group and the control group. Moreover, all landmark features were noted in the case of patients from the study group (26). On the other hand, Alhinti et al. assessed acoustic differences in the emotional speech of four dysarthric patients caused by cerebral palsy (1 person) or by Parkinson’s disease (3 persons) in comparison with 21 healthy individuals. The authors analyzed the speech rate (determined by the number of syllables spoken per time unit – calculated using a Praat script) and HNR (harmonic-to-noise ratio) of the dysarthric patients and contrasted them with healthy subjects. Furthermore, shimmer and jitter values were compared between female and male speakers (29). The study does not include an analysis of features such as performance time, which is the subject of our analysis.

Sechidis et al., as in our own research, conducted an objective assessment of speech through a machine learning modeling approach, however, in patients with Parkinson’s disease. Researchers used the Mixture-of-Experts (MoE) architecture to recognize speech-related emotions (30). The difference between the evaluation of our objective device and that of Sechidis et al. is that our device examines dysarthric disorders in the course of neurological diseases (MS in this case) based on the time of implementation of individual texts, without touching on the issue of emotionality of the statement. We also used longer texts for the study, making, in our opinion, the assessment of the disorder more objective.

To sum it up, it can be stated that our findings pave the way to a better understanding of speech characteristics in the group of MS patients with dysarthria and also indicate directions for the therapeutic process of dysarthric speech disorders. The examined aspects seem to be important due to the fact that the incidence of MS is increasing both nationally and globally. In addition, it should be emphasized that currently in Poland there are no objective tools for assessing speech disorders, adding a practical dimension to this study by introducing the first device of this type in our country.

## 5. Limitations

The study presents some limitations. First of all, our group of surveyed people with MS was practically homogeneous in terms of level of education, 83.33% had secondary education, therefore we were unable to analyze speech according to the level of education, which may have an impact on speech. In our research, we also did not analyze the type of work the subjects performed. Therefore, further research on a bigger group of MS patients is necessary to divide them according to their level of education and occupation. Secondly, the study included the assessment of mixed spastic-ataxic dysarthria only in the course of multiple sclerosis in patients aged 20 to 62. Therefore, further research into the matter is necessary to consider both dysarthric patients suffering from other illnesses, other types of dysarthria as well as other age groups, i.e., children, teenagers, and the elderly. Thirdly, the study is of preliminary character, aiming at evaluating whether the created objective tool is useful in detecting discrepancies in speech parameters between persons with speech disorders and control ones. It is essential to continue research in a larger group of patients aiming at validating the created device, comparing it with a test already in use and assessing its reliability and sensitivity. Additional studies should also include evaluating the effectiveness of therapeutic programs in terms of improving speech parameters.

## 6. Conclusion

The study showed statistically significant differences in the speaking speed in all analyzed speech samples, i.e., reading a poem, reading prose, and performing a diction exercise between people from the study group with MS with dysarthria and the both control groups: healthy controls and MS without dysarthria controls. Reading a poem, reading prose, and completing the diction exercise took more time in the dysarthria study group. Thus, the comparison between the groups in terms of sex and age showed disturbances in the analyzed samples in the study group. However, there were no significant differences in terms of sex and age, separately in the group of people with MS with dysarthria and in both control groups. The developed objective tool for speech analysis is useful in detecting differences in speech parameters, such as speed, between people with MS with dysarthria and healthy people and MS without dysarthria and can serve diagnostic purposes in clinical practice to improve the understanding of speech characteristics of MS patients with dysarthria, however, further research is needed to validate the created device.

## Data availability statement

The raw data supporting the conclusions of this article will be made available by the authors, without undue reservation.

## Ethics statement

The studies involving human participants were reviewed and approved by the Bioethical Committee at the University of Rzeszów (approval no. 3/01/2020). The patients/participants provided their written informed consent to participate in this study.

## Author contributions

WW: conceptualization and methodology. MP, WW, and AG: investigation, formal analysis, and writing—original draft preparation. HB-P, AG, and MD: data curation and writing—review and editing. WW and MP: project administration. All authors contributed to the article and approved the submitted version.

## Funding

The IT tool created in the course of the project run by BD CENTER Ltd.: Innovative IT tool aiding diagnostic process, prognosis and tracking of change dynamics in neurological patients – development work and its implementation’ No. RPPK.01.02.00-18-0004/20 co-financed by the European Regional Development Fund carried out within Regional Operational Programme of Podkarpackie Voivodeship 2014–2020 - Priority Axis 1: Competitive and Innovative Economy.

## Conflict of interest

WW and MP were employed by BD Center Ltd.

The remaining authors declare that the research was conducted in the absence of any commercial or financial relationships that could be construed as a potential conflict of interest.

## Publisher’s note

All claims expressed in this article are solely those of the authors and do not necessarily represent those of their affiliated organizations, or those of the publisher, the editors and the reviewers. Any product that may be evaluated in this article, or claim that may be made by its manufacturer, is not guaranteed or endorsed by the publisher.

## References

[1] Dobson R Giovannoni G. Multiple sclerosis - a review. Eur J Neurol. (2019) 26:27–40. doi: 10.1111/ene.13819 30300457

[2] Walton C King R Rechtman L Kaye W Leray E Marrie RA. Rising prevalence of multiple sclerosis worldwide: insights from the atlas of MS, third edition. Mult Scler. (2020) 26:1816–21. doi: 10.1177/1352458520970841, PMID: 33174475 PMC7720355

[3] Wnuk M Maluchnik M Perwieniec J Podwojcic K Szelag M Walkiewicz D. Multiple sclerosis incidence and prevalence in Poland: data from administrative health claims. Mult Scler Relat Disord. (2021) 55:103162. doi: 10.1016/j.msard.2021.103162, PMID: 34332458

[4] Oh J Vidal-Jordana A Montalban X. Multiple sclerosis: clinical aspects. Curr Opin Neurol. (2018) 31:752–9. doi: 10.1097/WCO.0000000000000622 30300239

[5] Compston A Coles A. Multiple sclerosis. Lancet. (2008) 372:1502–17. doi: 10.1016/S0140-6736(08)61620-7 18970977

[6] Kuo SH. Ataxia. Continuum (Minneap Minn). (2019) 25:1036–54. doi: 10.1212/CON.0000000000000753, PMID: 31356292 PMC7339377

[7] Morawska J Niebudek-Bogusz E Stasiołek M Świderek-Matysiak M Pietruszewska W. Speech pathology-specific questionnaire for persons with multiple sclerosis (SMS): adaptation, validation and preliminary assessment of the diagnostic potential. Mult Scler Relat Disord. (2021) 49:102796. doi: 10.1016/j.msard.2021.102796, PMID: 33540280

[8] Rusz J Benova B Ruzickova H Novotny M Tykalova T Hlavnicka J. Characteristics of motor speech phenotypes in multiple sclerosis. Mult Scler Relat Disord. (2018) 19:62–9. doi: 10.1016/j.msard.2017.11.007, PMID: 29149697

[9] Fazeli M Moradi N Soltani M Naderifar E Majdinasab N Latifi SM. Dysphonia characteristics and vowel impairment in relation to neurological status in patients with multiple sclerosis. J Voice. (2020) 34:364–70. doi: 10.1016/j.jvoice.2018.09.018, PMID: 30342799

[10] Merson RM Rolnick MI. Speech-language pathology and dysphagia in multiple sclerosis. Phys Med Rehabil Clin N Am. (1998) 9:631–41. doi: 10.1016/S1047-9651(18)30254-7, PMID: 9894114

[11] Chiara T Martin D Sapienza C. Expiratory muscle strength training: speech production outcomes in patients with multiple sclerosis. Neurorehabil Neural Repair. (2007) 21:239–49. doi: 10.1177/1545968306294737, PMID: 17351085

[12] De Pauw A Dejaeger E D'hooghe B Carton H. Dysphagia in multiple sclerosis. Clin Neurol Neurosurg. (2002) 104:345–51. doi: 10.1016/s0303-8467(02)00053-7 12140103

[13] Fischer MT Wimmer I Höftberger R Gerlach S Haider L Zrzavy T. Disease-specific molecular events in cortical multiple sclerosis lesions. Brain. (2013) 136:1799–815. doi: 10.1093/brain/awt110, PMID: 23687122 PMC3673462

[14] Klugman TM Ross E. Perceptions of the impact of speech, language, swallowing, and hearing difficulties on quality of life of a group of south African persons with multiple sclerosis. Folia Phoniatr Logop. (2002) 54:201–21. doi: 10.1159/000063194, PMID: 12169806

[15] De Biagi F Heikkola LM Nordio S Ruhaak L. Update on recent developments in communication and swallowing in multiple sclerosis. Int J MS Care. (2020) 22:270–5. doi: 10.7224/1537-2073.2020-023, PMID: 33424482 PMC7780699

[16] Duffy JR. Motor speech disorders: Substrates, differential diagnosis, and management. 3rd ed. Saint Louis, MO: Elsevier Mosby Press (2013).

[17] Guzy A. Voice emission diagnosis using a structured interview: practical implications. Logopaedica Lodziensia. (2019) 3:79–91. doi: 10.18778/2544-7238.03.06

[18] Olszewski J Nowosielska-Grygiel J. New diagnostic methods of evaluation of voice activity for phoniatrists’ and speech therapists’ needs. Logopaedica Lodziensia. (2017) 1:91–9. doi: 10.18778/2544-7238.01.08

[19] Knuijt S Kalf JG van Engelen BGM de Swart BJM Geurts ACH. The Radboud dysarthria assessment: development and Clinimetric evaluation. Folia Phoniatr Logop. (2017) 69:143–53. doi: 10.1159/000484556, PMID: 29393211

[20] Ziegler W Schölderle T Brendel B Risch V Felber S Ott K. Speech and nonspeech parameters in the clinical assessment of dysarthria: a dimensional analysis. Brain Sci. (2023) 13:113. doi: 10.3390/brainsci13010113, PMID: 36672094 PMC9856358

[21] Staiger A Schölderle T Brendel B Ziegler W. Dissociating Oral motor capabilities: evidence from patients with movement disorders. Neuropsychologia. (2017) 95:40–53. doi: 10.1016/j.neuropsychologia.2016.12.010, PMID: 27939368

[22] Pruszewicz A. Methods of voice organ examination. Adv Head Neck Surg. (2002) 1:3–25.

[23] El-Wahsh S Heard R Bogaardt H Kumfor F Ballard KJ. Variables associated with self-reported language impairment in multiple sclerosis: a regression analysis. Int J MS Care. (2021) 23:85–92. doi: 10.7224/1537-2073.2020-096, PMID: 33880085 PMC8047682

[24] Diederik P. Kingma J. B. Adam (2014). A method for stochastic optimization. Proceedings of the 3rd International Conference on Learning Representations (ICLR). 12:22.

[25] Noffs G Perera T Kolbe SC Shanahan CJ Boonstra FMC Evans A. What speech can tell us: a systematic review of dysarthria characteristics in multiple sclerosis. Autoimmun Rev. (2018) 17:1202–9. doi: 10.1016/j.autrev.2018.06.010, PMID: 30316992

[26] Hartelius L Elmberg M Holm R Lovberg AS Nikolaidis S. Living with dysarthria: evaluation of a self-report questionnaire. Folia Phoniatr Logop. (2008) 60:11–9. doi: 10.1159/000111799, PMID: 18057906

[27] Rodgers JD Tjaden K Feenaughty L Weinstock-Guttman B Benedict RH. Influence of cognitive function on speech and articulation rate in multiple sclerosis. J Int Neuropsychol Soc. (2013) 19:173–80. doi: 10.1017/S1355617712001166, PMID: 23058309 PMC5564302

[28] Liu CT Chen YS. Consonantal landmarks as predictors of dysarthria among English-speaking adults with cerebral palsy. Brain Sci. (2021) 11:1550. doi: 10.3390/brainsci11121550, PMID: 34942852 PMC8699804

[29] Alhinti L Christensen H Cunningham S. Acoustic differences in emotional speech of people with dysarthria. Speech Comm. (2021) 126:44–60. doi: 10.1016/j.specom.2020.11.005

[30] Sechidis K Fusaroli R Orozco-Arroyave JR Wolf D Zhang YP. A machine learning perspective on the emotional content of parkinsonian speech. Artif Intell Med. (2021) 115:102061. doi: 10.1016/j.artmed.2021.102061, PMID: 34001321

